# Comparative Evaluation and Correlation of Hyoid Bone Position, Airway Dimension, and Psychological Status in Class II Vertical and Horizontal Malocclusion Cases With Temporomandibular Disorder Compared to Class I Non-Temporomandibular Disorder Cases

**DOI:** 10.7759/cureus.68648

**Published:** 2024-09-04

**Authors:** Aathira Surendran, Pallavi Daigavane, Sunita Shrivastav, Ranjit Kamble, Abhishek D Sanchla, Lovely Bharti, Mrudula Shinde, Aditya V Pareek

**Affiliations:** 1 Orthodontics and Dentofacial Orthopedics, Sharad Pawar Dental College and Hospital, Wardha, IND; 2 Orthodontics and Dentofacial Orthopedics, Datta Meghe Institute of Higher Education & Research, Wardha, IND

**Keywords:** temporomandibular disorder (tmd), tmj disorders, psychological status, hyoid bone, tmd symptoms

## Abstract

Introduction

Temporomandibular disorder (TMD) involves discomfort and impaired function in the masticatory muscles and temporomandibular joint (TMJ), with a multifaceted etiology that includes biomechanical, neuromuscular, psychological, and biological factors. This research aims to assess and correlate the hyoid bone position, airway dimensions, and psychological status in class II Hz (horizontal) and Vt (vertical) malocclusion cases with TMD in contrast to non-TMD class I cases.

Methodology

This research was carried out at the Orthodontics Department, Sharad Pawar Dental College and Hospital, Sawangi, with consent obtained from the ethical committee. A total of 63 adult patients with class I, class II horizontal, and class II vertical malocclusions were selected. TMD was diagnosed using the Helkimo index, and patients were categorized accordingly. Psychological status was evaluated using the Warwick-Edinburgh scale, while the placement of hyoid bone and airway dimensions were assessed using lateral cephalograms. Statistical analysis involved descriptive and inferential statistics using SPSS version 27.0, with a significance level of p < 0.05.

Results

The research showed a noteworthy difference in the hyoid bone’s location, airway dimensions, and psychological status among the three groups. Class II TMD patients (both vertical and horizontal) exhibited higher hyoid bone positions and larger Go-Hy-Me angles (angle formed by the gonion-hyoid line and the hyoid-menton line) compared to class I patients. In addition, class II vertical TMD patients showed the most reduced airway dimensions. Psychological assessments indicated higher stress, anxiety, and depression levels in class II TMD patients, particularly in the vertical group, compared to class I non-TMD patients.

Conclusion

This study highlights the intricate relationships between hyoid bone position, airway dimensions, and psychological status in TMD patients. TMD patients present with hyoid bones positioned closer to the cranium and mandible, larger Go-Hy-Me angles, and reduced airway dimensions. Psychological distress exacerbates TMD symptoms, negatively impacting overall well-being and quality of life. Orthodontists should consider these interrelated factors when devising treatment plans to improve patient outcomes. Future longitudinal studies with larger samples and advanced imaging techniques are recommended to further elucidate these interactions.

## Introduction

The manifestations of temporomandibular disorder (TMD) encompass discomfort and impaired function in the masticatory muscles and the temporomandibular joint (TMJ). This condition is linked to a broad range of clinical symptoms, with its etiology being complex and involving various contributing elements such as biomechanical, neuromuscular, psychological, and biological factors. In addition, symptoms including sleep disturbances, airway blockage, and psychological discomfort have been associated with TMD [1].

The pharynx, cervical spine, cranium, and jaw are anatomically linked to the horseshoe-shaped hyoid bone through a network of ligaments and muscle attachments. The hyoid bone exhibits mobility during essential activities such as respiration, mastication, and articulation, all of which are under the influence of TMJ function [2]. Proper stomatognathic system function necessitates harmonious operation among the mandible, hyoid bone, and TMJ. Deviation in the position of the hyoid bone location may lead to discomfort in the jaw, neck, temporal region, and TMJ. Deviations in hyoid bone placement are capable of influencing TMJ dynamics and potentially contributing to TMD [3].

Studies have demonstrated a connection between the positioning of the hyoid bone and respiratory complications, indicating a notable correlation between lower hyoid bone placement and obstructive sleep apnea (OSA) [4]. Depending on whether the hyoid bone relocates upwards or downwards, alterations in its placement may either be advantageous or detrimental to individuals affected by OSA [5].

Individual, interpersonal, and environmental elements that impact a patient's capacity to operate adaptively are referred to as psychosocial factors. TMD is a physical and psychological illness that causes exhaustion, sleep problems, anxiety, and sadness [6]. Anxiety is more frequently reported in patients with TMDs compared to healthy control groups. In some individuals, psychological strain may specifically manifest as TMDs or orofacial pain symptoms. Patients with TMDs often experience pain that can impact their quality of life, sleep, and mood. Stimulation of the sympathetic nervous system and psychological issues, such as anxiety and depression, can place additional stress on the TMJ and masticatory muscles [7]. Moreover, contemporary research has validated the link between psychological dysfunctions and chronic pain. Therefore, assessing the psychological status of TMD patients can help orthodontists better understand the impact of TMD on patients’ overall well-being and tailor treatment plans accordingly [8].

Aim

This study aimed to assess, compare, and correlate the deviation in hyoid bone location, airway dimension, and psychological status among class II (horizontal (Hz) and vertical (Vt)) TMD cases and non-TMD class I cases.

Objective

The primary objective of this research was to determine deviation in hyoid bone location, airway dimension, and psychological status in class II (Hz and Vt) TMD cases in contrast to class I (Non-TMD) cases and correlate the variation in hyoid bone position, airway dimension, and psychological status with the skeletal pattern.

## Materials and methods

A total of 63 adult patients with class I, class II Vt, and class II Hz malocclusions were chosen from the orthodontic outpatient department (OPD) at Sharad Pawar Dental College in Sawangi, Wardha. This observational study, approved under number DMIHER(DU)/IEC/2023/1151 by the DMIHER Ethical Committee, was carried out at the Department of Orthodontics at Sharad Pawar Dental College and Hospital, Sawangi, with each participant's informed consent.

The study included adult patients who presented a complete set of permanent teeth and were grouped based on their diagnosis of class I malocclusion, class II (Vt) malocclusion with TMD, or class II (Hz) malocclusion with TMD. The participants in the study were a minimum of 18 years of age.

The study excluded cases of class II (Vt) without TMD, class II (Hz), or class III malocclusion. Furthermore, individuals with myalgia, a history of trauma or TMJ surgery, or myofascial pain dysfunction were not included in the research.

Method

The Helkimo index was employed to diagnose and classify patients with TMDs. Patients were segmented into three categories based on the arrangement of their cranium: class I, comprising cases without TMD; class II (Vertical), involving instances with TMD; and class II (Horizontal), encompassing cases with TMDs. The emotional state of the patients will be assessed using the Warwick-Edinburgh scale [9], while the positioning of the hyoid bone and airway will be evaluated using a lateral cephalogram.

Sample Size Calculation

The sample size calculation was conducted using the formula based on the mean difference: 

\[n1 = n2 = 2\frac{(Z_\alpha + Z_\beta)^2\sigma^2}{(\delta)^2}\],

where $Z_\alpha$ is 1.64, representing the type I error at 5% with a two-tailed test, and $Z_\beta$ is 0.84, corresponding to a power of 80%. The primary variable of interest is the hyoid triangle (C3-RGN distance in mm), with the reference article providing values for the TMD group (64.1 ± 8.6 mm) and the control group (70.6 ± 8.3 mm). The clinically relevant difference is 6.5 mm, and the pooled standard deviation is calculated as 8.45 mm.

Using these values, the sample size for each group was calculated as:

\[N1 = 2 * \frac{[(1.64 + 0.84)^2(8.45)^2]}{(6.5)^2} = 21\]

**Figure 1:**
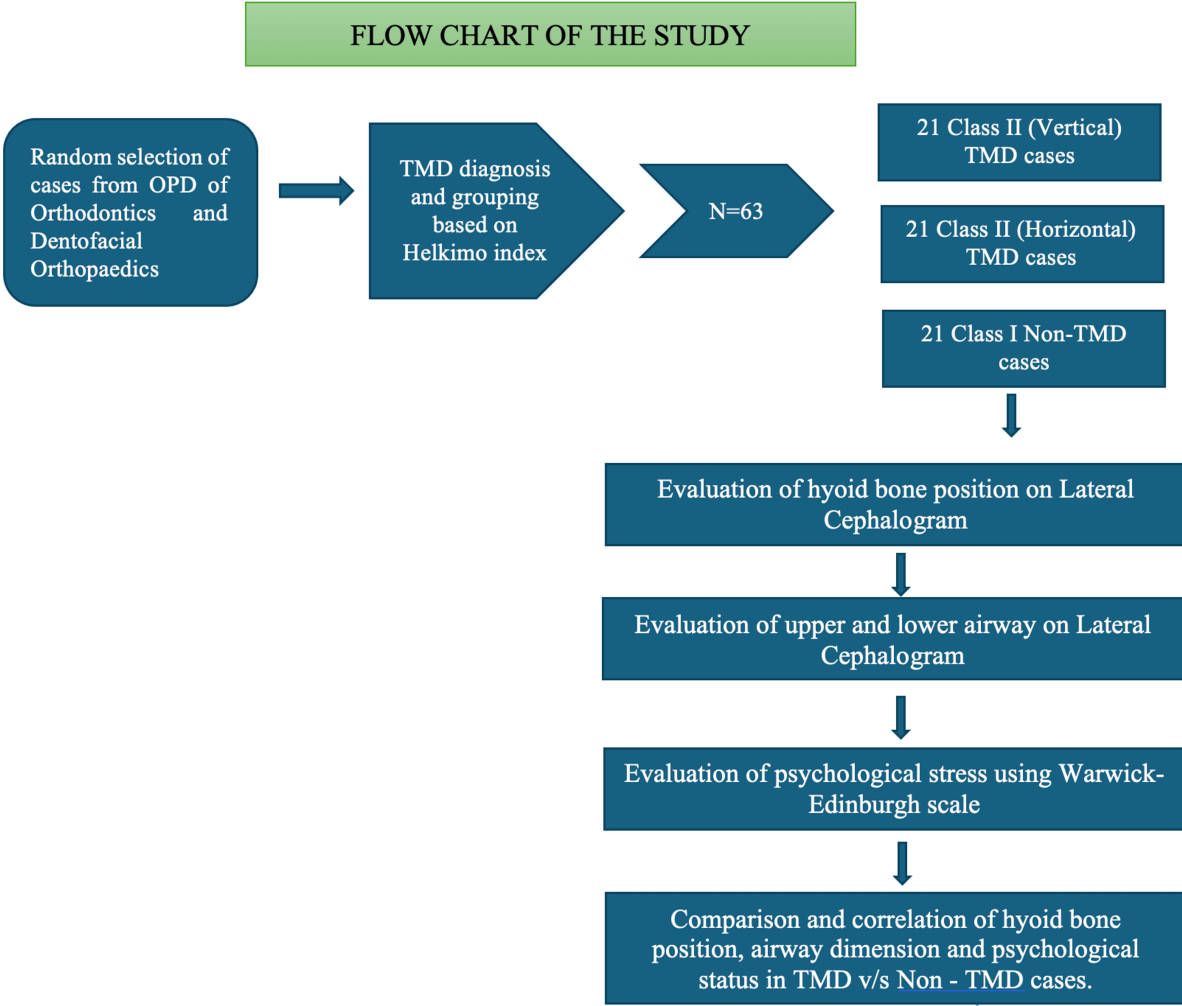
Outline of the study OPD: outpatient department, TMDL temporomandibular disorder

To account for the prevalence of severe cases at 0.95%, 15 patients were selected per group. Therefore, 21 patients were needed for each of the three groups, resulting in a total sample size of 63. The study will delineate three distinct groups based on the inclusion and exclusion criteria: Group A (control group) will include 21 class I (non-TMD) patients, Group B will consist of 21 class II (vertical) cases, and Group C will comprise 21 class II (horizontal) cases. The outline of the study design is depicted in Figure 1.

**Figure 2:**
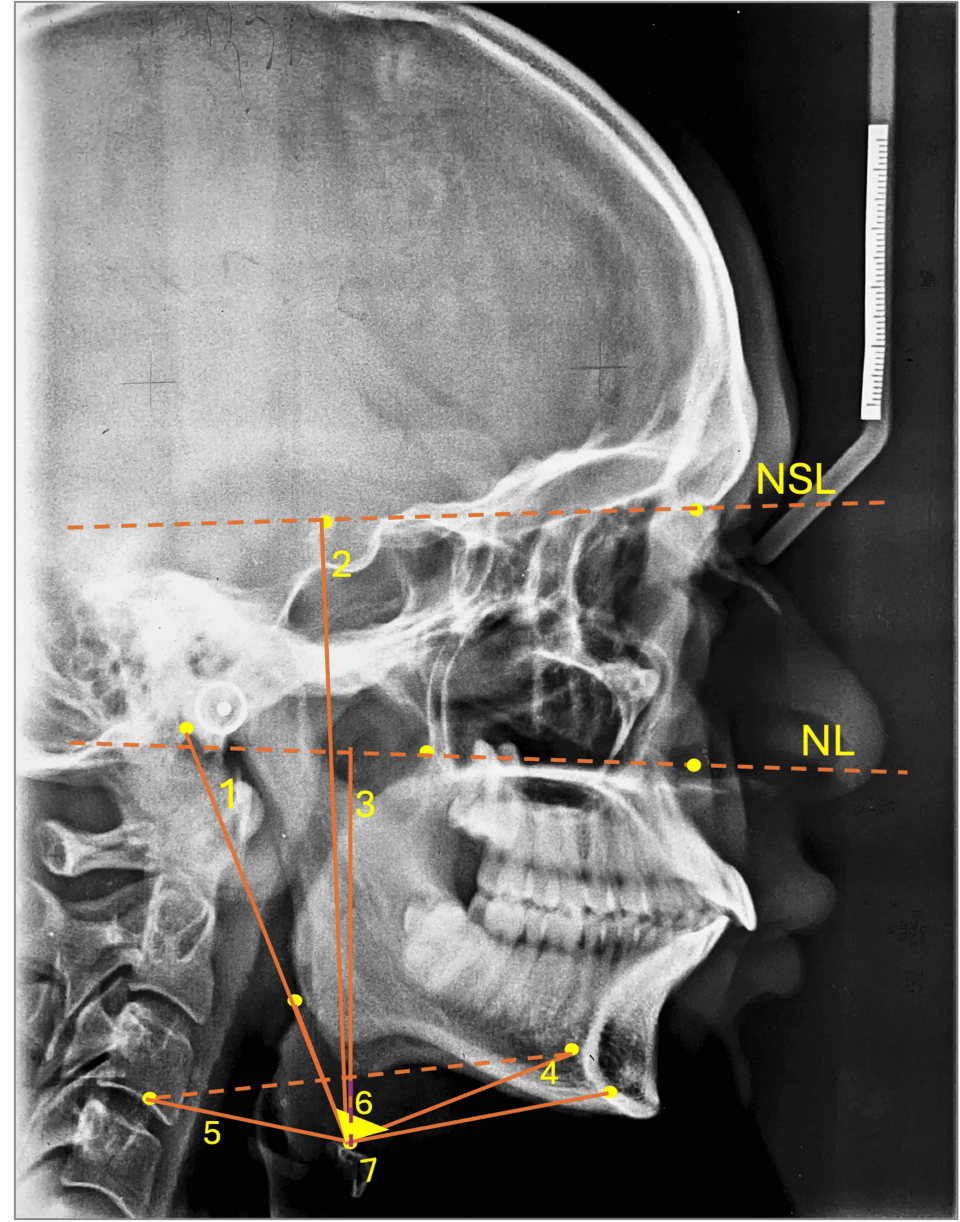
Variables of the hyoid bone position 1. Hy-Ba (mm), 2. perpendicular from Hy to NSL(mm), 3. perpendicular from Hy to NL(mm), 4. Hy- RGn(mm), 5. Hy-cv3ia(mm), 6. Hy to cv3ia-RGn, 7. Go/Hy/Me(^0^)

The study categorized the subjects into three distinct groups: Group 1 (average skeletal class), Group 2 (Vt skeletal class II), and Group 3 (Hz skeletal class II). In this study, both inferential and descriptive statistics were employed, encompassing the application of the Tukey test for multiple comparisons and one-way analysis of variance (ANOVA). The statistical analysis was executed using IBM SPSS Statistics for Windows, Version 27.0 (released 2021, IBM Corp., Armonk, NY), adhering to a predetermined significance threshold of p < 0.05. The principal objective of the study was to juxtapose the hyoid bone position, airway, and psychological states of patients exhibiting class II Vt and class II Hz TMD against those of individuals presenting class I non-TMD. Specifics on the cephalometric measurements, including angles and linear distances, are provided in Table 1 and Figure 2. 

**Table 1:** Cephalometric measurements (linear distances and angles) used in the study Hy- hyoidale, Ba- basion, NSL- nasion-sella line, NL- nasal line, Go- gonion, Me- menton, RGn (most protrusive point of retrognathion), cv3ia (most anteroinferior point on the third cervical vertebrae)

Hyoid bone position	
Hy-Ba (mm)	Hyoidale and basion's linear distance from one another.
Hy to NSL (mm)	Distance measured perpendicularly from the hyoidale to the Nasion-Sella line.
Hy to NL (mm)	Distance measured perpendicularly from the nasal line to the hyoidale
Go/Hy/Me (^0^)	The angle formed by the hyoidale, menton and gonion
Hy-RGn (mm)	Hyoidale and RGn’s linear distance from one another
Hy-cv3ia (mm)	Hyoidale and cv3ia’s linear distance from one another
Hy to cv3ia-RGn (mm)	Distance measured perpendicularly from the hyoidale and cv3ia-RGn plane

The Warwick-Edinburgh Mental Well-Being Scale (WEMWBS) is a validated 14-item questionnaire designed to assess psychological functioning and subjective well-being. All questions are positively worded, aiming to capture key elements of mental health. Each question is rated on a one- to five-point Likert scale. Scores on the WEMWBS range from a minimum of 14 to a maximum of 70. The WEMWBS is distinct from other mental health measures in that it lacks a defined threshold that would categorize the population into those with "good" and "poor" mental health [9].

Scoring

The overall scale score is derived by summing the scores of the 14 individual items in the WEMWBS. Each item is rated on a scale of 1 (never) to 5 (always) as indicated in Table 2.

**Table 2:** Warwick–Edinburgh Mental Well-Being Scale (WEMWBS) Self-assessment of emotional and mental well-being over the past two weeks: The participants were asked to indicate how often they have experienced each of the listed feelings or behaviors during the last two weeks by ticking the appropriate box, with 1 representing "None of the time" and 5 representing "All of the time."

Statements	None of the time	Rarely	Some of the time	Often	All of the time
I’ve been feeling optimistic about the future.	1	2	3	4	5
I’ve been feeling useful.	1	2	3	4	5
I’ve been feeling relaxed.	1	2	3	4	5
I’ve been feeling interested in other people.	1	2	3	4	5
I’ve had energy to spare.	1	2	3	4	5
I’ve been dealing with problems well.	1	2	3	4	5
I’ve been thinking clearly.	1	2	3	4	5
I’ve been feeling good about myself.	1	2	3	4	5
I’ve been feeling close to other people.	1	2	3	4	5
I’ve been feeling confident.	1	2	3	4	5
I’ve been able to make up my own mind about things.	1	2	3	4	5
I’ve been feeling loved.	1	2	3	4	5
I’ve been interested in new things.	1	2	3	4	5
I’ve been feeling cheerful.	1	2	3	4	5

## Results

Correlation of hyoid bone position in class II vt and class II hz TMD patients versus non-TMD patients

**Figure 3:**
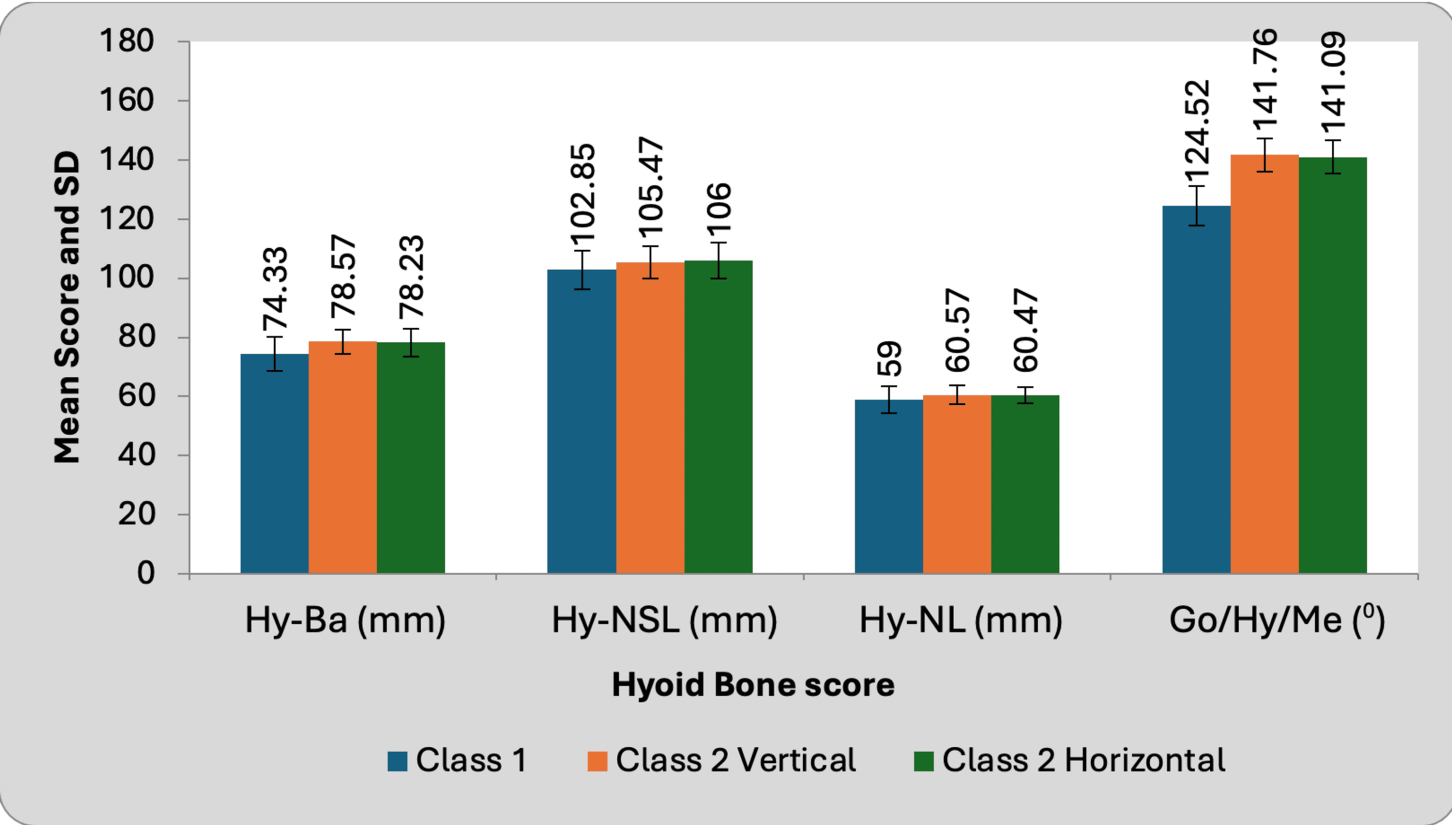
Correlation of hyoid bone position in class II vertical, class II horizontal TMD patientz versus non-TMD patients TMD- temporomandibular disorder

The orientation of the hyoid bone demonstrated noteworthy divergence among individuals diagnosed with class II Hz TMD, class II Vt TMD, and non-TMD class I patients, as substantiated by the outcomes of the Hy-Ba and Go-Hy-Me assessments. Upon contrasting the TMD cohort with the control group, it was discerned that the former manifested augmented hyoid angles and diminished Hy-NL, Hy-Ba, and Hy-NSL parameters. The gauging of hyoid bone positioning within the confines of class II Hz TMD, class II Vt TMD, and non-TMD class I cohorts is delineated in Table 3 and Figure 3 for elucidation.

**Table 3:** Correlation of the hyoid bone position in class II vertical, class II horizontal TMD patient versus non-TMD patients S- significant, NS- non-significant, statistical test- multiple comparison: Tukey test, TMD- temporomandibular disorder

Variables	Group		Mean difference	Std. error	p-value	95% confidence interval	
						Lower bound	Upper bound
Hy-Ba (mm)	Class 1	Class 2 Vertical	-4.23	1.50	0.018, S	-7.86	-0.61
		Class 2 Horizontal	-3.90	1.50	0.032, S	-7.53	-0.27
	Class 2 Vertical	Class 2 Horizontal	0.33	1.50	0.973, NS	-3.29	3.95
HY-NSL (mm)	Class 1	Class 2 Vertical	-2.61	1.86	0.345, NS	-7.09	1.85
		Class 2 Horizontal	-3.14	1.86	0.219, NS	-7.62	1.33
	Class 2 Vertical	Class 2 Horizontal	-0.52	1.86	0.957, NS	-5.00	3.95
HY – NL (mm)	Class 1	Class 2 Vertical	-1.57	1.12	0.347, NS	-4.26	1.12
		Class 2 Horizontal	-1.47	1.12	0.392, NS	-4.17	1.21
	Class 2 Vertical	Class 2 Horizontal	0.09	1.12	0.996, NS	-2.60	2.79
Go/Hy/Me (°)	Class 1	Class 2 Vertical	-17.23	1.87	0.0001, S	-21.75	-12.72
		Class 2 Horizontal	-16.57	1.87	0.0001, S	-21.08	-12.05
	Class 2 Vertical	Class 2 Horizontal	0.66	1.87	0.933, NS	-3.84	5.18

The data present the Hy-Ba readings for the three distinct groups. The average readings for Groups 1 and 2 are 74.33, 78.57, and 78.23, with Group 1 exhibiting the lowest average. A thorough statistical analysis reveals significant variations among the groups. It is noteworthy that both class II Vt and class II Hz exhibit comparable hyoid bone (Hy) positions in relation to basion (Ba), and these positions are marginally higher than those of class I. Furthermore, the average hyoid bone position with respect to the nasion-sella line (NSL) is relatively higher in class II (both Vt and Hz) than in class I, with respective averages of 102.85, 105.47, and 106. The hyoid bone position relative to the nasion line (NL) is quite similar across all three classes, with class 2 Vt and Hz showing very slight increases compared to class 1 with a mean of 59 for class I, 60.57 for class II Vt, and 60.47 for class II Hz. The angle between the gonion (Go), hyoid bone (Hy), and menton (Me) is significantly larger in class 2 (both Vt and Hz) compared to class 1, indicating a notable difference in the spatial relationship of these anatomical landmarks with a mean of 124.52 for class I, 141.76 for class II Vt, and 141.09 for class II Hz.

Comparison of the airway dimension in all three groups

The data present the measurements of the upper and lower airways for the three different groups. The mean of the upper airway for Group 1 was 16.47, 13.14 for Group 2, and 14.42 for Group 3. The statistical analysis indicates notable differences among the groups. Group 1 has the greatest average upper airway measurement at 16.47. Class 2 Vt has the lowest mean upper airway measurement at 13.14. Class 2 Hz falls in between with a mean upper airway measurement of 14.42. The upper airway is the widest in Class 1 and narrowest in Class 2 Vt, indicating potential differences in airway space that could have clinical implications, such as breathing or sleep issues. While the mean of lower airway for Group 1 was 12.38, 10.33 for Group 2, and 11.33 for Group 3. Similar to the upper airway, the lower airway is widest in Class 1 and narrowest in Class 2 Vt, again suggesting differences in airway space that could impact respiratory function. On comparing airway dimensions in class II Vt TMD patients and class II Hz TMD patients, there was a significant difference found in both groups, and it was significantly more in the class II Vt TMD group. The airway dimension in class II Vt TMD, class II Hz TMD, and non-TMD patients is delineated in Table 4.

**Table 4:** Comparison of airway dimensions in all the three groups S- significant, NS- non-significant, statistical test- multiple comparison: Tukey test

Variables	Group		Mean difference	Std. error	p-value	95% confidence interval	
						Lower bound	Upper bound
Upper airway (mm)	Class 1	Class 2 Vertical	3.33	0.36	0.0001,S	2.45	4.21
		Class 2 Horizontal	2.04	0.36	0.0001,S	1.16	2.93
	Class 2 Vertical	Class 2 Horizontal	-1.28	0.36	0.003,S	-2.16	-.40
Lower airway (mm)	Class 1	Class 2 Vertical	2.04	0.39	0.0001,S	1.10	2.98
		Class 2 Horizontal	1.04	0.39	0.025,S	0.10	1.98
	Class 2 Vertical	Class 2 Horizontal	-1.00	0.39	0.034,S	-1.93	-.06

Comparison of the psychological status in class II hz TMD patients and class II vt TMD patients with non-TMD class I patients

**Figure 4:**
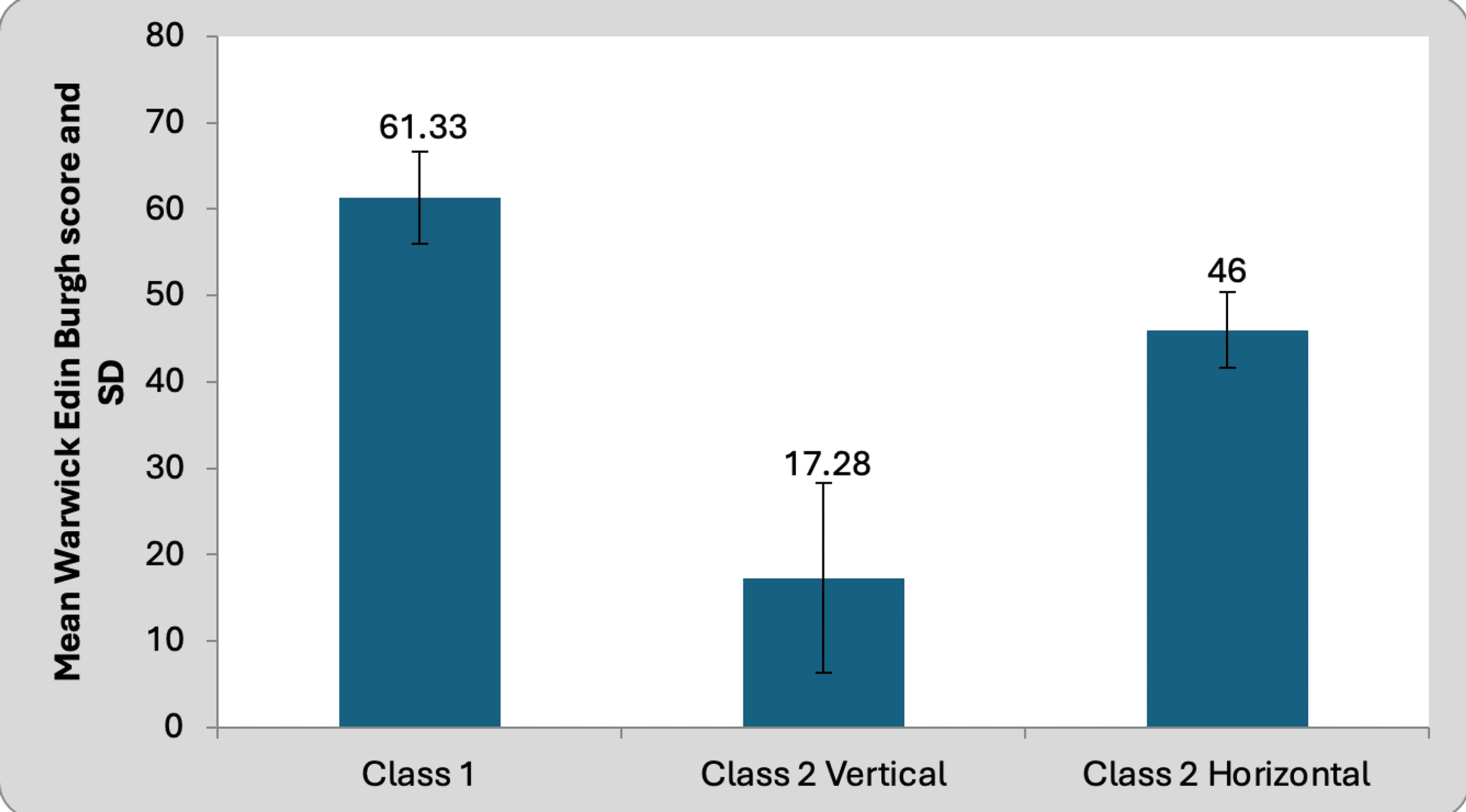
Comparison of psychological status in class II vertical TMD patients and class II horizontal TMD patients with class I non-TMD patients

The data illustrate the Warwick-Edinburgh results for the three distinct groups. Groups 2 and 3 displayed average scores of 17.28 and 46.00, respectively, while Group 1 exhibited an average score of 61.33. The psychological status of class II vertical TMD, class II horizontal TMD, and non-TMD patients is delineated in Table 5 and Figure 4 for elucidation.

**Table 5:** Comparison of psychological status in class II vertical TMD patients and class II horizontal TMD patients with class I non-TMD patients S- significant, statistical test- multiple comparison: Tukey test

Variables	Group		Mean difference	Std. error	p-value	95% confidence interval	
						Lower bound	Upper bound
Warwick-Edinburgh	Class 1	Class 2 Vertical	44.04	2.31	0.0001, S	38.49	49.60
		Class 2 Horizontal	15.33	2.31	0.0001, S	9.77	20.88
	Class 2 Vertical	Class 2 Horizontal	-28.71	2.31	0.0001, S	-34.27	-23.15

A statistical analysis reveals significant variations among the groups. Furthermore, there exists a substantial variance in the psychological condition between class II Vt and class II Hz TMD patients when compared to class I non-TMD patients.

## Discussion

The present study aimed to comparatively evaluate and correlate the hyoid bone position, airway dimension, and psychological status among patients with class II Hz TMD, class II Vt TMD, and non-TMD class I. Our evaluation of TMD severity utilized the Helkimo index [10], comprising a dysfunction component for objective symptoms and an amnestic component for subjective symptoms. The amnestic component included a questionnaire pertaining to TMJ noises, jaw stiffness, and discomfort in the masticatory muscles. The dysfunction component involved a clinical assessment of mandibular motions, masticatory muscle palpation, and evaluation of clicking and luxation. The final grade was determined using a 25-point dysfunction scale.

Our results indicated distinct differences in the hyoid bone position among the groups. In class II Vt TMD patients, the hyoid bone was positioned more inferiorly and posteriorly compared to both class II Hz TMD and non-TMD class I individuals. This inferior and posterior positioning could be attributed to the vertical growth pattern, often resulting in a more elongated facial structure and altered craniofacial dynamics. By contrast, class II Hz TMD cases showed a relatively more anterior hyoid position, potentially due to the forward growth pattern commonly seen in these patients. Understanding these positional differences is crucial, as the hyoid bone plays a significant role in airway patency and head posture. Numerous research studies have delved into the correlation between the position of the hyoid bone and TMD. Zhou et al. conducted a study in which they observed that adult TMJ OA (temporomandibular joint osteoarthritis) patients exhibited hyoid bone positions closer to the skull and mandible with shorter distances between C3 and retrognathion compared to healthy individuals [11]. In a separate study, Ekici et al. found that TMD patients, compared to controls, had shorter distances between the hyoidale and the sella-nasion line and between the hyoidale and basion [12]. However, findings from a study by Andrade et al. did not indicate notable differences in the hyoid bone position between TMD patients and controls [13]. Consequently, the current data are insufficient to conclusively establish a link between the positioning of the hyoid bone and the occurrence of TMDs.

Upon observation, Ekici and Camci noted that out of a total of 113 individuals, 55 of whom had TMD and 58 comprised the healthy control group, and TMD patients exhibited larger Go-Hy-Me angles and had hyoid bones positioned closer to the skull [12]. In addition, a separate study revealed that TMD patients displayed larger Go-Hy-Me angles, and their hyoid bones were positioned in closer proximity to the jaw and skull compared to other groups [14]. Our findings demonstrate a notable increase in the angle between the gonion (Go), hyoid bone (Hy), and menton (Me) in class 2 (both vertical and horizontal) in comparison to Class 1. This strongly indicates a correlation between the positioning of the hyoid bone and TMD, potentially resulting in a lowered tongue position and a constricted airway.

Airway dimension analysis revealed that class II Vt TMD patients had significantly reduced airway dimensions compared to the other groups. This reduction can be linked to the posterior positioning of the hyoid bone, which may contribute to a narrower airway space. In our study, class II Hz TMD patients, while having a compromised airway compared to class I bon-TMD, still demonstrated better airway dimensions than the vertical TMD group. In a 2006 research study conducted by Freitas et al., it was found that patients exhibiting vertical development patterns and class I and class II malocclusions displayed notably diminished upper pharyngeal airway dimensions in comparison to individuals with normal growth patterns and class I and class II malocclusions [5].

Psychological factors are both causes and symptoms of TMD, creating a vicious cycle. Studies, such as those by Yap AU et al. in 2021, have identified stress as a major factor in triggering or exacerbating TMDs [15]. Good mental well-being decreases the chances of experiencing painful and capsular TMD symptoms, whereas overall anxiety increases them, as noted by Yap AU et al. in 2022 [16]. Psychological assessments indicated that TMD patients, irrespective of the subclass, had higher levels of anxiety and stress compared to class I non-TMD individuals. However, class II Vt TMD patients exhibited the highest psychological distress, possibly due to the combined effects of TMD pain, compromised airway, and altered craniofacial structure affecting their quality of life. These findings align with previous studies that have highlighted the psychological burden associated with chronic pain conditions like TMDs.

In 2006, Gamiero et al. established a correlation between symptoms of temporomandibular joint disorder, anxiety, and tense muscles. Their findings revealed that 16.58% of the participants exhibiting TMD symptoms displayed signs of anxiety, while 26.71% showed symptoms of sadness. Another study assessing the psychological well-being of TMD patients reported that 47.6% experienced moderate to severe nonspecific physical symptoms (somatization) and 39.8% exhibited moderate to severe depression [17].

Patients with more severe TMD exhibit heightened levels of psychological distress and a diminished Oral Health-Related Quality of Life (OHRQoL). The experience of TMD is influenced by various psychological components such as behavioral symptoms (e.g., bruxism), emotions (e.g., stress, worry, and sadness), cognitive behavior, and aspects of long-term memory [7]. These psychological factors directly impact an individual's overall well-being and QoL. Most TMD patients are teenagers or young adults, a critical period for development. TMD during this phase can affect productivity, concentration, and social relations, leading to impaired QoL both personally and professionally [18]. This is in line with our results, which show the impact of psychological factors in TMD patients because of increased stress and anxiety due to decreased sleep and bruxism, which impacts overall well-being [19]. Since previous studies have not correlated the hyoid bone position, airway difficulty, and psychological distress with TMD, this study provides valuable information for orthodontists to develop effective treatment plans that address the complex interactions between TMD, airway obstruction, and psychological distress. By adopting a comprehensive approach to TMD treatment, orthodontists can enhance patient outcomes and quality of life [20].

The study found significant correlations between hyoid bone position, airway dimension, and psychological status. Specifically, a more inferiorly positioned hyoid bone was associated with reduced airway dimensions and higher psychological distress. These correlations highlight the multifaceted nature of TMD and the need for a comprehensive diagnostic approach that includes airway evaluation and psychological assessment. Clinicians should be aware of these interrelationships to tailor treatment plans that address not only the mechanical aspects of TMD but also the airway and psychological components.

The primary limitation of this study stems from its observational design, which precludes the demonstration of a causal association between hyoid location and TMDs. Additional long-term investigations and three-dimensional assessments are necessary to comprehensively explore this relationship and the correlation between the bilateral location of the hyoid bone and TMJ. Additional limitations include a small participant sample and the use of two-dimensional cephalometric radiographs. Future studies should use 3D imaging for more comprehensive analysis.

## Conclusions

This study underscores the multifaceted nature of TMDs, illustrating the intricate relationships between hyoid bone position, airway dimensions, and psychological status. Our findings highlight that TMD patients often present with hyoid bones positioned closer to the cranium and mandible, accompanied by larger Go-Hy-Me angles, which correlate with reduced airway dimensions. Moreover, psychological distress, manifesting as increased stress, anxiety, and depression, exacerbates TMD symptoms, thereby negatively impacting patients' overall well-being and quality of life. This comprehensive approach emphasizes the necessity for orthodontists to consider these interrelated factors when devising treatment plans, ultimately aiming to improve patient outcomes and life quality. However, the observational nature of this study limits the ability to establish causality, necessitating future longitudinal research with larger samples and advanced three-dimensional imaging to further elucidate these complex interactions.
